# The Cynomolgus Macaque Natural History Model of Pneumonic Tularemia for Predicting Clinical Efficacy Under the Animal Rule

**DOI:** 10.3389/fcimb.2018.00099

**Published:** 2018-04-04

**Authors:** Tina Guina, Lynda L. Lanning, Kristian S. Omland, Mark S. Williams, Larry A. Wolfraim, Stephen P. Heyse, Christopher R. Houchens, Patrick Sanz, Judith A. Hewitt

**Affiliations:** ^1^National Institute of Allergy and Infectious Diseases, National Institutes of Health, Bethesda, MD, United States; ^2^Mergus Analytics, LLC, Jericho, VT, United States; ^3^Biomedical Advanced Research and Development Authority, Department of Health and Human Services, Washington, DC, United States

**Keywords:** *Francisella tularensis*, pneumonic tularemia, cynomolgus macaque, non-human primate, animal model, Animal Rule, Animal Model Qualification

## Abstract

*Francisella tularensis* is a highly infectious Gram-negative bacterium that is the etiologic agent of tularemia in animals and humans and a Tier 1 select agent. The natural incidence of pneumonic tularemia worldwide is very low; therefore, it is not feasible to conduct clinical efficacy testing of tularemia medical countermeasures (MCM) in human populations. Development and licensure of tularemia therapeutics and vaccines need to occur under the Food and Drug Administration's (FDA's) Animal Rule under which efficacy studies are conducted in well-characterized animal models that reflect the pathophysiology of human disease. The Tularemia Animal Model Qualification (AMQ) Working Group is seeking qualification of the cynomolgus macaque (*Macaca fascicularis*) model of pneumonic tularemia under Drug Development Tools Qualification Programs with the FDA based upon the results of studies described in this manuscript. Analysis of data on survival, average time to death, average time to fever onset, average interval between fever and death, and bacteremia; together with summaries of clinical signs, necropsy findings, and histopathology from the animals exposed to aerosolized *F. tularensis* Schu S4 in five natural history studies and one antibiotic efficacy study form the basis for the proposed cynomolgus macaque model. Results support the conclusion that signs of pneumonic tularemia in cynomolgus macaques exposed to 300–3,000 colony forming units (cfu) aerosolized *F. tularensis* Schu S4, under the conditions described herein, and human pneumonic tularemia cases are highly similar. Animal age, weight, and sex of animals challenged with 300–3,000 cfu Schu S4 did not impact fever onset in studies described herein. This study summarizes critical parameters and endpoints of a well-characterized cynomolgus macaque model of pneumonic tularemia and demonstrates this model is appropriate for qualification, and for testing efficacy of tularemia therapeutics under Animal Rule.

## Introduction

*Francisella tularensis* is a highly infectious Gram-negative bacterium that is the etiologic agent of tularemia in animals and humans. Infections with highly virulent *F. tularensis* strains are lethal in 30–60% of individuals infected by the inhalation route if not treated with antibiotics, and *F. tularensis* strains have been weaponized for potential use as a biothreat agent (Stuart and Pullen, 1945; Hornick, 1998; Dennis et al., 2001). For these reasons, *F. tularensis* has been designated a Tier 1/Category A select agent by the Centers for Disease Control (CDC) and the National Institutes of Allergy and Infectious Diseases (NIAID), part of the National Institutes of Health (NIH). The natural incidence of pneumonic tularemia in the United States is very low (CDC, 2013). Hundreds of people have contracted tularemia in outbreaks in other countries, primarily in Eurasia (Dahlstrand et al., 1971; Christenson, 1984; Tarnvik et al., 1996; Ohara et al., 1998; Perez-Castrillon et al., 2001; Reintjes et al., 2002; Meka-Mechenko et al., 2003; Celebi et al., 2006). However, due to the sporadic nature of these outbreaks, it is not feasible to conduct clinical efficacy testing of tularemia vaccines and therapeutics in human populations. In the United States, tularemia medical countermeasures (MCM) efficacy studies can be conducted in accordance with the Animal Rule [21 CFR Parts 314 Subpart I and 21 CFR Part 601 Subpart H, Approval of New Drugs When Human Efficacy Studies Are Not Ethical or Feasible, and Food and Drug Administration (FDA, 2009, 2015)]. Drug or vaccine licensure under the Animal Rule requires availability of at least one well-characterized animal model displaying pathophysiology similar to disease in humans (FDA, 2009, 2015). Animal models are important components of preparedness as they are used for testing candidate MCM in response to public health emergencies (e.g., anthrax, Ebola, pandemic influenza).

FDA's Animal Model Qualification (AMQ) Program (FDA, 2017) under the Drug Development Tools Program is a regulatory pathway for qualification of animal models to be used for MCM efficacy testing and approval under the Animal Rule. A qualified animal model is product-independent and may be used for efficacy testing of multiple investigational drugs for the targeted disease or condition. A Tularemia Animal Model Qualification Working Group (AMQ WG) was established to integrate and analyze natural history studies to seek qualification of the cynomolgus macaque (*Macaca fascicularis*) model of pneumonic tularemia as a Drug Development Tool with the FDA. The intent for use of this model is demonstrating the efficacy of therapeutics for treatment and/or post-exposure prophylaxis of pneumonic tularemia. This model may be further refined for use in efficacy testing of tularemia vaccines.

Among non-human primates (NHPs), rhesus and cynomolgus macaques, African Green monkeys, and marmosets have been used in tularemia studies (Lyons and Wu, 2007). Recent studies (Glynn et al., 2015, and data described in this manuscript) demonstrated that the infectious dose and disease pathophysiology in cynomolgus macaques challenged with aerosolized *F. tularensis* strain Schu S4, closely resemble human pneumonic tularemia caused by *F. tularensis* subsp. *tularensis* (Type A) strains, the most severe and lethal form of the disease. It is assumed that therapeutics and vaccines that show efficacy in the NHP model that reflects the most severe form of tularemia should also be efficacious against less severe forms of tularemia. *F. tularensis* Schu S4 is a highly virulent strain which caused febrile disease and other symptoms in volunteers after inhalation of <20 colony forming units (cfu) (Saslaw et al., 1961). It has been weaponized and used in human volunteer challenge studies and animal model development worldwide since the 1940's (Dennis et al., 2001; Lyons and Wu, 2007). Historically, rhesus macaques have been used in tularemia research, but currently available rhesus macaque colonies have been more resistant to infection with aerosolized Schu S4 when compared to cynomolgus macaques and humans (median lethal dose, LD_50_ ~10^5^ cfu for rhesus, and LD_50_ ~20 cfu for cynomolgus macaques; Saslaw et al., 1961; Glynn et al., 2015 and unpublished). For these reasons, the cynomolgus macaque was chosen for development of the pneumonic tularemia model in NHPs.

This manuscript describes the progression of pneumonic tularemia in cynomolgus macaques based on data collected in five natural history studies and one antibiotic efficacy study in which animals were exposed to aerosolized *F. tularensis* Schu S4. These previously unpublished studies were performed to develop and establish a NHP model of pneumonic tularemia for efficacy testing of therapeutics and vaccines at three sites including Battelle Biomedical Research Center (BBRC, West Jefferson, OH), Lovelace Respiratory Research Institute (LRRI, Albuquerque, NM), and the United States Army Medical Research Institute for Infectious Diseases (USAMRIID, Frederick, MD). Two natural history studies also included additional pathogenesis progression (serial pathology) study arms in which animals were euthanized for gross pathology examination and tissue analysis at specific days following challenge with *F. tularensis* to evaluate disease progression. Although the objectives and endpoints of all studies were similar, certain aspects of the individual study protocols (e.g., subject study inclusion and exclusion criteria) varied somewhat as the understanding of the model progressed over time. For example, one early study included groups of animals exposed to three target doses of 50, 500, and 5,000 cfu aerosolized *F. tularensis*, while all other studies targeted a 1,000 cfu exposure dose. Mortality, average time to death, average time to fever onset, average interval between fever and death, bacteremia, clinical signs, necropsy findings, and histopathology from the studies were analyzed and form the basis for the proposed cynomolgus macaque model parameters and endpoints. Telemetry data from four natural history studies and one antibiotic efficacy study was used for analyses of the core body temperature and for calculating onset of fever and hypothermia in individual animals and across studies. Tularemia AMQ WG recommendations on critical model parameters that are appropriate for use of this model for testing of MCMs under Animal Rule (see section Results) are based on the analysis of data from all six studies and comparison of the results with human cases of pneumonic tularemia from published literature. The manuscript describes suggested model inclusion and exclusion criteria, critical model parameters, model for calculation of onset of fever as the physiologic trigger for initiation of therapy, and model endpoints. Data collected and analyzed to date support the conclusion that signs of pneumonic tularemia in cynomolgus macaques exposed to a target dose of 1,000 cfu (range 300–3,000 cfu) aerosolized *F. tularensis* Schu S4, under the conditions described herein, and human pneumonic tularemia cases are highly similar. Challenge doses lower than 300 cfu Schu S4 resulted in a wider range of symptoms, disease clinical course, survival rates and pathology outcomes, as was also observed in previous studies (Glynn et al., 2015, and data described in this manuscript). Analyses also support the use of fever onset as the physiologic trigger for initiation of therapeutic interventions during MCM efficacy testing.

## Materials and methods

### Test animals and study inclusion criteria

Cynomolgus macaques (*M. fascicularis*) of ~2 to ~7 years of age that originated from Indochina were obtained from commercial vendors in the United States. Animals were from colonies that originated with wild caught animals and were raised at the vendor facilities. Thirty-eight (38) female and forty (40) male animals were included in five natural history studies (Studies 1, 2, 3, 4, and 6; see Table 1). Two natural history study protocols (Studies 1 and 4) also included serial pathology study arms in which gross pathology and histopathology were evaluated on specific days after challenge with *F. tularensis* (Table 1), starting with Day 2 after challenge. Animals in the antibiotic efficacy study (Study 5) were administered either a vehicle or antibiotic solution intravenously (i.v.) after fever onset was determined. Because animals in the antibiotic efficacy study were treated differently than in natural history studies following fever onset, Study 5 data were used only for analyses of body temperature and fever onset, but not for analysis of survival time and biomarkers of disease progression. In natural history studies (Studies 1 through 4 and 6, Table 1), females were 2.3–7.7 years old and weighed 2.3–7.2 kg; males were 2.5–6.5 years old and weighed 2.6–7.4 kg. Nineteen animals (10 female, 9 male) included in the antibiotic efficacy study (Study 5, Table 1) were 3.0–7.0 years of age and weighed 2.5–4.0 kg. Macaques were prescreened and negative for prior exposure to *Mycobacterium tuberculosis, Klebsiella pneumoniae, Salmonella* sp., *Shigella* sp., simian immunodeficiency virus (SIV), simian retrovirus 1 and 2 (SRV1 and 2) and simian T-lymphotropic virus-1 (STLV-1), Macacine Herpesvirus 1 (Herpes B virus), and Simian Retrovirus (SRV1 and SRV2) prior to receipt at testing facilities. After arrival at testing facilities, animals were quarantined for at least 30 days before acceptance onto the studies. Previous exposure of animals to *F. tularensis* was examined by determination of anti-*F. tularensis* antibody titers using a tube agglutination test with *F. tularensis* antigen (BD, Franklin Lakes, NJ) at BBRC; or by ELISA, which measured binding of serum antibodies to the heat-inactivated whole cell preparation of *F. tularensis* subsp. *holarctica* Live Vaccine Strain (LVS) at LRRI. Animals showing no humoral response to *F. tularensis* were included in the studies. In addition to these screens, macaques were also screened for previous exposure to *Trypanosoma cruzi* (via PCR and serology testing) and for infection with *Plasmodium* species in Study 5 (Table 1). Prior to release from quarantine, animals underwent a complete physical examination by a clinical veterinarian that included evaluation of a complete blood count (CBC), serum chemistry screen, and fecal ova and parasite determination. Only animals that were considered clinically healthy by veterinarian, including the above test results, were admitted onto the studies.

**Table 1:** Summary of studies.

**Study number**	**Study design**	**Animals in natural history study arm**	**Animals in pathogenesis progression study arm**	**Study site**	**Target challenge dose (cfu)**	**Data shown in figures and tables**
1	Natural history	6 M, 6 F	8 M, 8 F	LRRI	1,000	Figures 2–6, Figure S1
						Tables 2, 3, Table S1
2	Natural history	4 M, 5 F	na	BBRC	1,000	Figures 1–5, Figure S1
						Tables 2, 3, Table S1
3	Natural history	5 M, 5 F	na	BBRC	1,000	Figures 2–5, Figure S1
						Tables 2, 3, Table S1
4	Natural history	4 M, 4 F	8 M, 8 F	LRRI	1,000	Figures 2–5, Figure S1
						Tables 2, 3, Table S1
5	Antibiotic efficacy, placebo control	5 M, 5 F (placebo)4 M, 5 F (drug)	na	BBRC	1,000	Figures 3–5 Table 2, Table S1
6	Natural history, dose ranging	16 M, 12 F	na	USAMRIID	50, 500, 5,000	Figure 2 Table 3, Table S1

*Cfu, colony forming units; M, male; F, female; na, not applicable; ST, Supporting Table; SF, Supporting Figure; LRRI, Lovelace Respiratory Research Institute; BBRC, Battelle Biomedical Research Center; USAMRIID, the United States Army Medical Research Institute for Infectious Diseases*.

All available information for each animal as well as all pre-study activities (i.e., telemetry placement surgery, catheter placement surgery, antibiotic treatment, etc.) were recorded in individual animal case report forms (CRFs). Once an animal was placed onto a study, all individual animal information was captured in each laboratory's data systems and was presented in the study report in both individual and summary data formats. The CRF (animal receipt to study assignment) and the study data (from the time of animal study assignment to post-life assessments) comprised the totality of the information available for each individual animal before and throughout the study.

### Ethics statement

All studies presented in this manuscript were approved by the responsible institution's Institutional Animal Care and Use Committee (IACUC), and research was conducted in compliance with the Animal Welfare Act and other federal statutes and regulations relating to animals. Experiments involving animals adhered to principles stated in the Guide for the Care and Use of Laboratory Animals from the National Research Council. Studies were performed at institutions which are fully accredited by the Association for Assessment and Accreditation of Laboratory Animal Care International (AAALAC). Studies were performed under IACUC approved protocol numbers FY-09-012 (Study 1), 1026-G607612 (Study 2), 2741-G607612 (Study 3), FY-14-048 (Study 4), 3195-100050134 (Study 5), and AP09-002 (Study 6). Animal health prescreening and study inclusion/exclusion criteria are provided above. Macaques were pair housed up to about 1 week preceding telemetry surgery after which they were individually housed in stainless steel cages on racks equipped with automatic watering systems that met the specifications of The Guide for the Care and Use of Laboratory Animals, and the Animal Welfare Regulations (AWR's). Environmental humidity, temperature, and light/dark cycles (12-h each) in were controlled and monitored. Animals were fed commercially available fixed-formula diets formulated specifically for NHPs to provide the proper balance of nutrients, with additional nutritional supplementation (e.g., extra vegetables, fruit, PrimaLac, Ensure). To promote and enhance the psychological well-being, the macaques underwent enrichment according to the institutional Standard Operational Protocols (SOPs). After exposure, macaques were observed by laboratory personnel thrice daily with clinical signs and body weights monitoring. Analgesics buprenorphine (0.01 mg/kg), ketamine (20 mg/kg) were used prior to telemetry transponder implantation surgery. Buprenorphine (0.01 mg/kg), and in some cases flunixin meglumine (2 mg/kg), were used twice daily for 3 days after the surgery. Macaques were anesthetized with Telazol (1–6 mg/kg) prior to aerosol exposure and euthanasia. Isoflurane 3–5% was used for anesthesia induction and 0.5–3% for maintenance during inhalation exposure. Animals were euthanized when moribund or at the end of the study following the American Veterinary Medical Association (AVMA) accepted methods of euthanasia.

### *F. tularensis* challenge strain history and provenance

*F. tularensis* Schu S4 is a highly virulent strain which caused febrile disease and other symptoms in volunteers after inhalation of <20 cfu (Saslaw et al., 1961) and has been used in many historic studies in animal models (Lyons and Wu, 2007). SchuS4 was also administered in a low dose respiratory challenge study in humans investigating the safety and efficacy of the LVS of *F. tularensis* (Saslaw et al., 1961).

Aerosolized Schu S4 was used in the studies described in this manuscript. Source of challenge material was NIAID Schu S4 submaster cell bank [“Sublot 1,” stored at the Biodefense and Emerging Infections Research Resources Repository (BEI) under NR-10492], used for the preparation of challenge material. NR-10492 cell bank was manufactured following Good Manufacturing Practice (GMP) procedures by direct dilution of GMP-manufactured NIAID Schu S4 Master Cell Bank (BEI NR-28534), and characterized for its viability (cfu/ml); Gram stain, colony morphology, identity, purity, median lethal dose (LD_50)_ in BALB/c mice inoculated subcutaneously (s.c.), and LD_50_ in New Zealand White Rabbits inoculated intranasally (i.n.), intravenously (i.v.), and s.c. The Master Cell Bank (BEI NR-28534) was originally propagated from the cell bank Lot No. 623-42 manufactured at the Salk Institute (Swiftwater, PA) in 1986. Cell bank Lot No. 623-42 was derived from a Schu S4 lot obtained from USAMRIID. Genomic sequences of NR-28534 and NR-10492 are publicly available at NCBI (accession numbers PRJNA270247 and PRJNA217349, respectively). Genome sequence annotation and analysis of NIAID cell banks NR-28534 and NR-10492 showed a single nucleotide polymorphism (SNP) in genomic DNA at location 1,767,864 of the published reference Schu S4 genome (GenBank number AJ749949.2) which is located in intergenic region between open reading frames FTT_1698c (predicted formate dehydrogenase) and FTT_r08 (predicted 5S ribosomal RNA). NIAID cell banks NR-28534 and NR-10492 contain two copies of *Francisella* pathogenicity island (FPI) and are highly virulent in Fischer rats when administered by aerosol (LD_50_ <1 cfu, Hutt et al., 2017).

### *F. tularensis* challenge material preparation, delivery, and route of administration

*F. tularensis* Schu S4 was stored and used in Biosafety level 3 (BSL-3) laboratories following institutional SOPs and federal regulations for work with select agents. Animals were exposed to a target dose of 1,000 cfu aerosolized Schu S4 in aerosol particles of 1–3 microns mass median aerodynamic diameter (MMAD) in four natural history studies (Studies 1–4) and an antibiotic efficacy study (Study 5). Natural history Study 6 (Table 1) included three groups of animals, each exposed to target dose of 50, 500, or 5,000 cfu aerosolized Schu S4. For preparation of challenge material, individual Schu S4 colonies that were first grown on glucose cysteine blood agar (GCBA) at 37°C (at BBRC and LRRI) or the thawed material from the vials of bacterial stock stored at −80°C (at USAMRIID) were inoculated in liquid culture media for further propagation. Enriched Mueller Hinton II Broth supplemented with 0.1% glucose, and 2% IsoVitaleX, pH 7.0 was used at BBRC and USAMRIID, while Chamberlain's broth (Chamberlain, 1965) was used for growth of *F. tularensis* at LRRI. Bacterial cultures were grown with aeration at 37°C until mid-log or early stationary phase of growth, after which they were suspended to a specific concentration (cfu/ml) in Brain Heart Infusion Broth (BHIB) at BBRC and LRRI, or enriched Mueller Hinton Broth (MHB) at USAMRIID to reach the desired target nebulizer mixture concentrations prior to aerosolization. Nebulizer mixtures used in animal exposures were analyzed by Gram stain, colony morphology, purity, and viability by plating 10-fold dilutions onto GCBA plates.

Prior to challenge with *F. tularensis*, macaques were anesthetized and placed into the exposure chamber inside a Class III biological safety cabinet (BSC). *F. tularensis* Schu S4 was aerosolized by a 3-jet Collison nebulizer and delivered via a head-only inhalation exposure chamber in the BSC. Aerosol samples were collected from the exposure chamber using an all glass impinger (AGI) containing BHIB supplemented with anti-foam. Bacterial concentrations in the nebulizer and AGI were determined by spreading diluted samples on agar media. The bacterial suspension in the nebulizer was enumerated before and after aerosol generation. All plate cultures were visually inspected for colony morphology consistent with *F. tularensis*, and for lack of contamination. The temperature, relative humidity, and aerosol particle sizes were monitored during each exposure. The aerosol particle size distribution was determined based on samples collected from the exposure chamber and measured using an Aerodynamic Particle Sizer (APS) Spectrometer. Body plethysmography was used for measurement of the individual animal tidal volume, total accumulated tidal volume, and minute volume. The duration of the aerosol exposure was based upon an estimated Schu S4 concentration (cfu/ml) in aerosol determined in earlier studies and a cumulative minute volume measured for individual animals. Methods for the consistent delivery of the target challenge dose of *F. tularensis* were qualified during pre-challenge studies with *F. tularensis* Schu S4 aerosols and no animals, to calculate the spray factor necessary to achieve target doses of *F. tularensis* based on a fixed starting concentration of bacteria.

### Clinical observations and histopathology

Animals were monitored for signs of disease including activity, body weight, morbidity, and mortality after the challenge for up to 14 days in Studies 2, 3, and 4, up to 28 days in studies 1 and 6, and up to 35 days after the challenge in the antibiotic efficacy Study 5. Telemetry transponders were implanted to measure physiologic parameters including core body temperature, respiration rate, systolic/diastolic pressure, and heart rate starting at 7–14 days before the challenge with *F. tularensis* and throughout the study. Core body temperature was measured in all studies, while respiration rate, systolic/diastolic pressure, and heart rate were measured in four of five natural history studies. Telemetry data were collected once for at least 30 s every 15 min. Body temperature data were summarized by hourly averages. Blood was collected for determination of bacteremia by plating on agar in all studies, and for hematology and clinical chemistry. Bacteremia and bacterial organ burdens were determined by plating on Chocolate II Agar or Thayer-Martin agar at 37°C. Organ burdens were typically determined by analysis of *F. tularensis* cell counts in organ lesions that were identified in course of gross pathology examination. Lesioned tissue was homogenized and plated on agar. The qPCR assay was used to detect bacterial DNA in animal blood in Studies 3 and 5. Briefly, nucleic acids were isolated from 100 μL whole blood samples on an EasyMAG instrument (bioMérieux, Durham, NC) and eluted into a final volume of 40 μL. The purified nucleic acid samples were quantified as a number of DNA gene copies present in the samples using qPCR with *F. tularensis tul4* specific DNA primers (forward primer GCAGGTTTAGCGAGCTGTTCTAC; reverse primer ATGATGCAAAAGCTTCAGCTAAAG) and a minor groove binding probe (CTAGGGTTAGGTGGCTCT). The samples were analyzed on an ABI 7900HT instrument (Applied Biosystems, Carlsbad, CA) and the results were analyzed using Sequence Detection Systems (SDS) software. A linear regression was fit to the reference standard curve with cycle threshold (C_T_) as the predictor and the logarithm of gene copies as the response.

Animals were humanely euthanized when moribund or at the end of the study. A complete necropsy was performed on all animals. External surfaces of the body, orifices, and the contents of the cranial, thoracic, and abdominal cavities were examined and all necropsy findings were recorded in descriptive terms including locations(s), size, shape, color, consistency, and number. Protocol-specified tissues were collected and organ bacterial burdens were evaluated from portions of tissues collected at necropsy, typically including spleen, liver, lungs, heart, brain, and various lymph nodes. All remaining tissues were fixed in 10% neutral buffered formalin. The fixed tissues were trimmed, embedded in paraffin, sectioned, and stained with hematoxylin and eosin for microscopic examination. An independent review of microscopic slides from these animals was performed by a blinded NIAID pathologist, to apply consistent terminology to the microscopic evaluation of tissues from animals in the natural history studies conducted at BBRC, LRRI, and USAMRIID.

### Body temperature model, fever onset determination, and hypothermia determination

Retrospectively after all studies were completed, body temperature recorded by telemetry (Studies 1–5; Study 6 had incomplete data) was modeled to evaluate a standardized approach to determining onset of fever; a similar framework was evaluated for determining morbidity, through onset of hypothermia. Hourly temperature readings during the period before exposure to the pathogen (“pre-exposure”) were modeled over time of day (24-h time transformed to a (0, 1] scale, i.e., midnight = 0, …, 600 h = 6/24 = 0.25, …, 2300 h = 23/24 = 0.9583) in the generalized additive models (GAM) framework. The smoothing function was a cyclic cubic spline to enforce smoothness over the midnight boundary. The data structure was accommodated through mixed effects: subject (nested in study and challenge date) and optionally study or challenge date (nested in study; Studies 1 and 5 included subsets of 6–10 subjects challenged on different dates 6–11 days apart) were included in the models as random effects on the intercept. Thus, the models comprise a GAM fixed effects component depicting the estimated population characteristic circadian rhythm of body temperature and a random effects component representing variation in baseline temperature due to subject, study, and challenge date. Model fitting was implemented using the gamm function in the *mgcv* package in R; both Akaike's Information Criterion (AIC) and likelihood ratio tests were considered for model selection (Wood, 2017). AIC balances model complexity and fit with lower AIC-values indicating better support. AIC is reported as AIC differences (ΔAIC) with the best-supported model having ΔAIC = 0.00 and other models having positive ΔAIC. Models within 2 AIC units of the best-supported model (ΔAIC < 2.00) are considered to have good support while those more than 2 AIC units back (ΔAIC ≥ 2.00) are considered to have poor support. Likelihood ratio tests are used only for nested pairs of models (Burnham and Anderson, 2003). Model predictions were appended to the full data set (i.e., pre- and post-exposure data) and differences from subject-specific predictions were taken as estimates of temperature elevation (or depression). Fever onset was determined as the first instance when temperature elevation was ≤1.5°C over two or more consecutive hourly averages. Similarly, hypothermia was determined as the first instance when temperature depression was ≤−1.5°C over two or more consecutive hourly averages. Time to fever was analyzed using linear mixed effects models (Pinheiro and Bates, 2000) with candidate fixed effects predictors inhaled dose, weight, age (all centered and rescaled), and sex; inference was based on model selection using AIC and likelihood ratio tests. Study was included as a random effect. Time of hypothermia was simply described and not subject to any hypothesis tests.

## Results

### Clinical signs and survival

**Figure 1:**
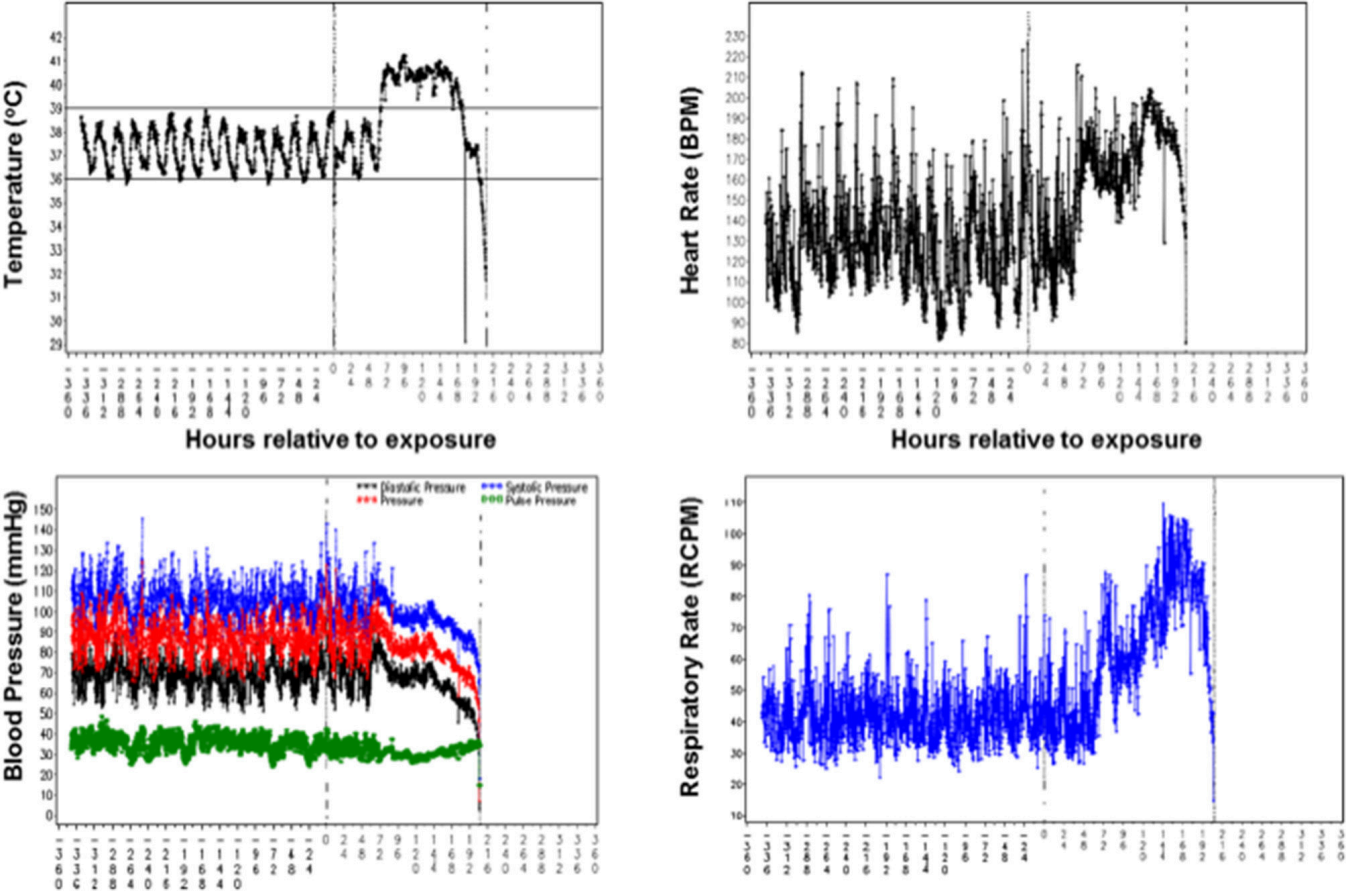
Telemetry data of the core body temperature, heart rate, blood pressure, and respiratory rate recorded for a cynomolgus macaque exposed to 758 cfu *F. tularensis* in Study 2 (Table 1). Values on X axes in each graph represent time in hours before and after challenge with *F. tularensis* Schu S4 at 0 h.

Clinical signs of pneumonic tularemia in animals exposed to aerosolized *F. tularensis* Schu S4 typically started on Day 2 or 3 after challenge and included hunched posture, followed by lethargy, coughing, weakness, loss of appetite and weight loss, lower activity, labored breathing, and in some cases respiratory distress. Normal core body temperature followed a diurnal pattern immediately after the challenge until an increase over the baseline was observed in all animals starting on Day 2 or 3 (see an example in upper left in Figure 1). Hypothermia was often observed as animals succumbed to disease. Average respiratory rates remained near baseline levels for 2–3 days after challenge, and then increased above the baseline with an upward trend until macaques succumbed (lower right in Figure 1). Average animal heart rates remained near baseline during the first 2 days after challenge. Then heart rates increased to significantly greater than baseline average and were sustained during the next several days (upper right in Figure 1). Systolic and diastolic blood pressures remained steady until Day 3 after challenge when both began to decrease slightly. Their rate of decrease accelerated around Day 6, continuing downward until death about 3 days later (lower left in Figure 1).

An increase in total circulating white blood cells (WBC) typically started on Day 2 after challenge and peaked on Day 4 followed by a decrease and falling below baseline starting on Day 6 after challenge (Figure S1). The increase in WBC counts was due to a pronounced neutrophilia. Lymphocyte levels dropped precipitously below the normal range by Day 3–4 and remained low as the disease progressed. A marked increase in the acute-phase C-reactive protein (CRP), a marker of inflammation, was observed in all animals, typically starting on Day 2 (Figure S1). CRP levels remained elevated until death. Serum biomarkers of tissue damage, lactate dehydrogenase (LDH), hepatic damage biomarkers aspartate aminotransferase (AST) and alanine aminotransferase (ALT), and kidney damage biomarker blood urea nitrogen (BUN) steadily increased with progression of tularemia, with significant increases in values over the pre-challenge baseline typically starting on Days 4 to 6 for LDH, Day 6 for AST and ALT, and Day 6 and later for BUN (not shown).

**Figure 2:**
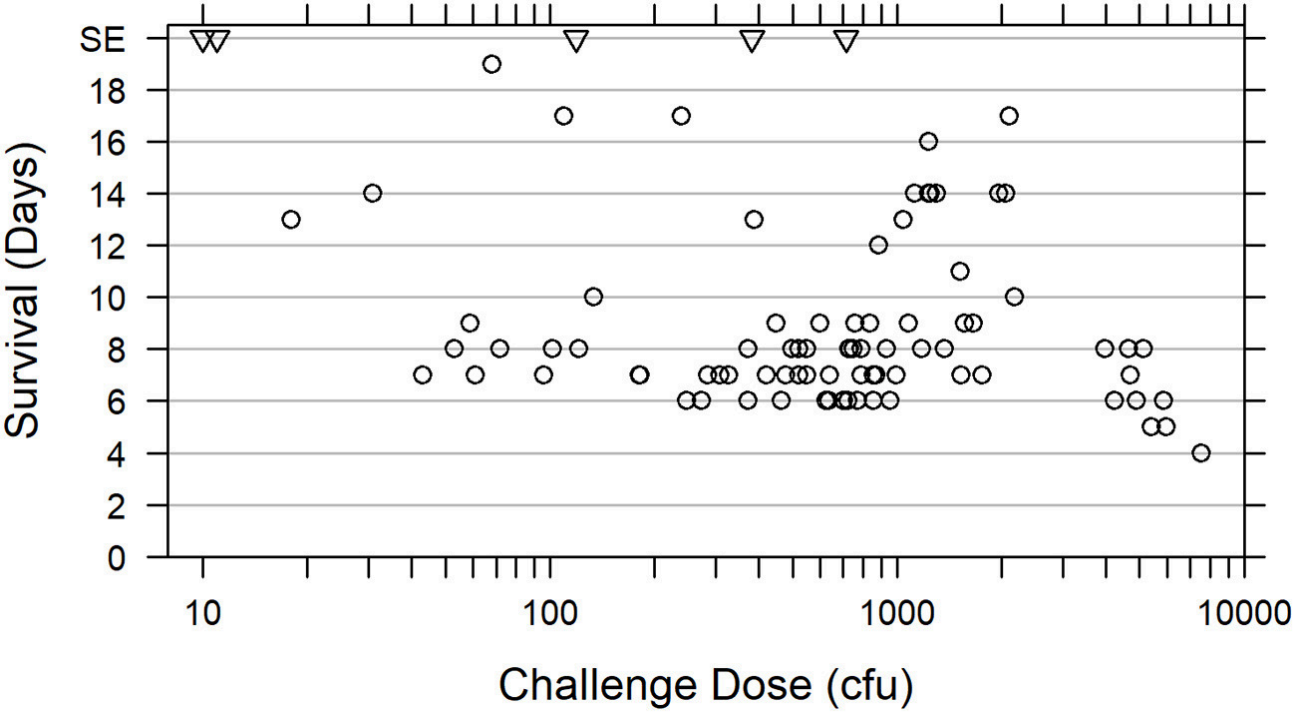
Relationship between survival of cynomolgus macaques and *F. tularensis* Schu S4 challenge dose. Survival data are presented for 73 cynomolgus macaques challenged with 18 to 7,550 cfu aerosolized *F. tularensis* Schu S4 in five natural history studies (Studies 1 through 4 and Study 6, Table 1) (open circles). Data for two animals from the pathogenesis progression arm of Study 4 which succumbed prior to scheduled euthanasia are also included. Five animals that survived to the end of the study (SE) after exposure to 10, 11, 119, 382, or 714 cfu in four different studies, are also represented (triangles).

Survival of animals challenged with doses ranging 18–7,550 cfu *F. tularensis* Schu S4 in five natural history studies (Studies 1 through 4 and 6, Table 1) is shown in Figure 2. Among animals that were challenged with ~300–3,000 cfu *F. tularensis* Schu S4, 44 out of 46 (95.7%) succumbed on Days 6 through 14, with two animals, each on a separate study, surviving the challenge. Challenge doses lower than 300 cfu Schu S4 resulted in a wider range of symptoms, disease clinical course, survival rates, and pathology outcomes, as it was also observed in previous studies (Glynn et al., 2015). A subset of animals challenged with doses above 3,000 cfu demonstrated faster disease progression and succumbed in 8 days or less (Figure 2 and Glynn et al., 2015). The severe pneumonic tularemia disease model in which animals are exposed to doses above 3,000 cfu aerosolized *F. tularensis* would have a very short timeline between onset of disease and moribund state, with a narrow window for initiation of successful therapeutic treatment and demonstration of drug efficacy. Based on these results, Tularemia AMQ WG proposes a target challenge dose of 1,000 cfu aerosolized *F. tularensis* Schu S4 with 300–3,000 cfu tolerated dose range for the cynomolgus macaque model of pneumonic tularemia.

### Bacteremia and bacterial organ burden

The presence of *F. tularensis* Schu S4 in blood in animals in the five natural history studies was examined after plating on agar. Positive cultures were found in 32 out of 38 animals that were challenged with 300–3,000 cfu aerosolized Schu S4 on Study Days 2–8 (Table S1). Bacteremia has not been consistently detected on specific days after the challenge in these studies and was not correlated with the Schu S4 doses. The commonly used method of plating blood on agar plates is not highly sensitive for detection of bacteremia in models of tularemia. BBRC has developed and validated a qPCR method for measuring circulating bacterial genomes in the cynomolgus macaque based on amplification of a segment of the *F. tularensis* Schu S4 gene *tul4*. The qPCR assay was able to detect bacterial genomes in blood 1–4 days earlier when compared to the agar plating method in Study 3 (Table S1). In addition, the qPCR method detected bacterial genomes in blood of three animals which were not bacteremic based on agar plating results in this study (Table S1).

*F. tularensis* was also detected by agar plating in various organs including lungs, spleen, liver, mandibular and thoracic lymph nodes, and kidney in all challenged animals, with lungs showing the highest burdens of up to ~10^8^ −10^9^ cfu *F. tularensis*/mg tissue in individual animals (not shown).

### Model of baseline core body temperature

Fever was successfully used as a therapeutic trigger in human studies in which subjects were challenged with *F. tularensis* Schu S4 (Saslaw et al., 1961). A comprehensive analysis of core body temperature data recorded by telemetry was done to evaluate a standardized approach to determining onset of fever. Telemetry transponders were implanted to measure core body temperature starting at 7–14 days before the challenge with *F. tularensis* Schu S4 until the end of the study. Telemetry data from five natural history studies and one antibiotic efficacy study were analyzed to estimate baseline circadian rhythm of body temperature (see section Methods). Baseline core body temperature in one natural history study (Study 6) was markedly more variable than what was observed in the other studies (variance of residuals 0.351 compared to 0.146 or less for the other five studies), and telemetry recording in several animals in this study failed. The inconsistency in body temperature recordings that were the norm for other studies reported herein, and telemetry failure in several animals was likely an outcome of unsuccessful telemetry transponder surgery in Study 6. Due to lack of consistent baseline core body temperature data, animal body temperatures from this study were excluded from the remainder of the analysis. Data from the other five studies (four natural history studies, Studies 1 through 4, and one antibiotic efficacy study, Study 5) supported modeling random effects of subject nested in study but not challenge date. The models that included challenge date or excluded study had Akaike's Information Criterion differences (Δ AIC) = 1.9 to 4.7 (Table 2). Furthermore, likelihood ratio tests indicated study is a significant random effect (Model 2 vs. 4, test statistic 6.70, *p* = 0.01) but challenge date is not (Model 1 vs. 2, test statistic 0.00003, *p* ≈ 1).

**Table 2:** Model selection for the random effects portion of the smooth model fit to pre-challenge data.

**Model number**	**Random effects**	**Extra df**	**nlL**	**ΔAIC**
1	Study, challenge date, subject	3	6,720.09	2.00
2	Study, subject	2	6,720.09	0.00
3	Challenge date, subject	2	6,721.05	1.91
4	Subject	1	6,723.44	4.70

*Extra degrees of freedom (df) refers to the number of random effects. Negative log-likelihood (nlL) reflects goodness of fit with smaller values indicating better fit. AIC differences (ΔAIC) indicate the best-supported model having ΔAIC = 0.00, models with ΔAIC < 2.00 considered to have good support, and those with ΔAIC ≥ 2.00 considered to have poor support (Burnham and Anderson, 2003)*.

**Figure 3:**
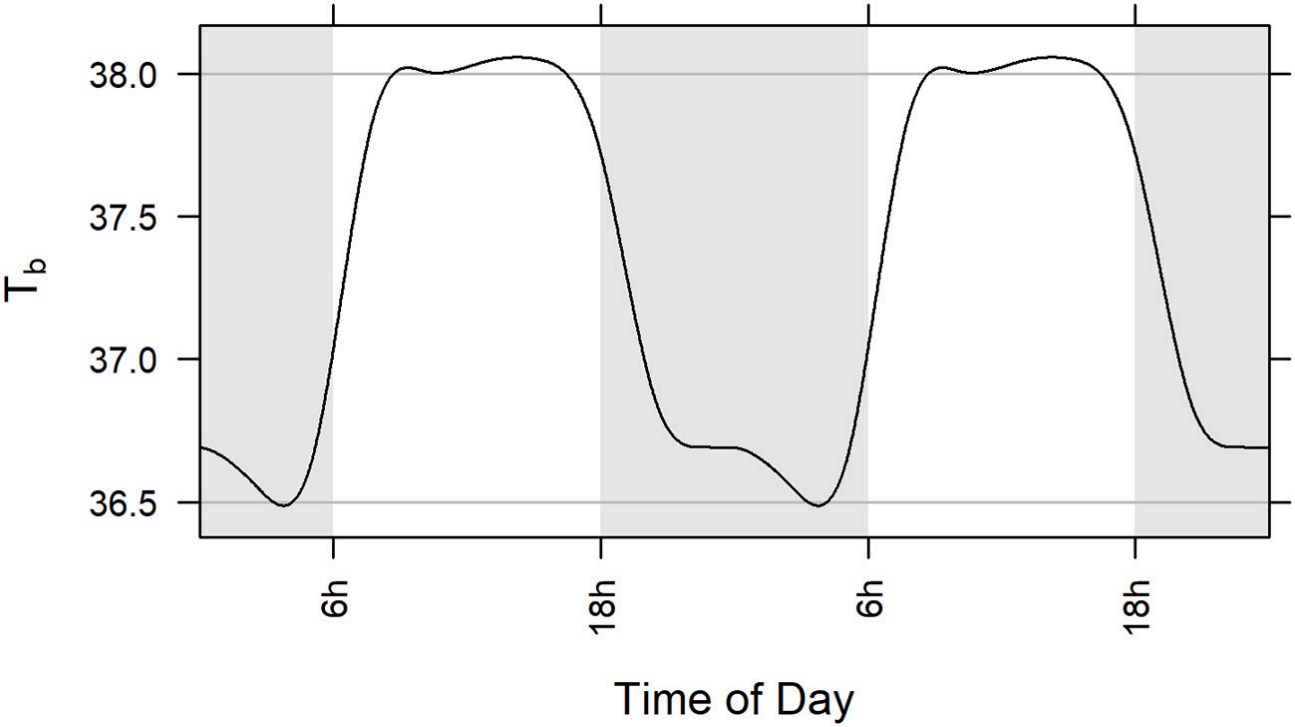
Population level model (GAM) of circadian rhythm of body temperature in five studies illustrated over two 24-h cycles. Model is based on baseline core body temperature data of all subjects challenged with aerosolized *F. tularensis* in Studies 1, 2, 3, 4, and 5 (Table 1). Gray lines at 36.5 and 38.0°C represent a range of 1.5°C. Statistical methods used are described in section Materials and Methods.

The GAM portion of the model was a parsimonious representation of baseline (pre-exposure) body temperature in the five included studies (adjusted *R*^2^ = 0.664). Among the subjects across the five studies, modeled body temperature rose to about 38.0°C during the day and dropped to about 36.5°C at night (Figure 3). The standard deviation of the innermost residuals from the fitted model was 0.32°C implying that observed temperature was expected to be within 1.5°C of the subject-specific prediction about 99.99% of the time. Note that defining fever as two consecutive hourly readings ≥1.5°C higher than the subject-specific prediction means that the probability of falsely determining fever is considerably <0.01%.

There were five instances in the pre-exposure period (two instances for one animal in Study 3 and three instances for one animal in Study 5) when hourly body temperature (T_b_) was elevated 1.5°C or more above subject-specific baseline predictions but none occurred over consecutive hours or even within the same day; there were no instances when T_b_ was depressed 1.5°C or more below subject-specific predictions before exposure to *F. tularensis*.

### Determination of fever onset

Among the 57 subjects included in this analysis (Studies 1 through 5, Table 1) that were challenged with Schu S4, all exhibited fever as defined above (core body temperature elevation ≥1.5°C over two or more consecutive hourly averages) (Table S1). Fifty-four (54) subjects had computed fever onset between 40 and 70 h after challenge, one exhibited fever 26 h after challenge, and two others exhibited fever about 80 h after challenge. Mean time to fever across all studies was 57.3 h (standard deviation 8.6 h). Approximately 20% of the variance in time to fever was attributable to differences among studies and random effects coefficients (study-specific estimates of time to fever) ranged from 52.8 to 61.8 h. The 95% confidence interval for the fixed effect coefficient of that model was (52.4, 62.2). Fever onset was typically concurrent with or immediately followed clinical signs of disease in challenged animals. Fever preceded detection of bacteremia and was detected more often than bacteremia, similar to observations in humans with pneumonic tularemia. Therefore, fever is a more consistent biomarker of disease in pneumonic tularemia when compared to bacteremia.

**Figure 4:**
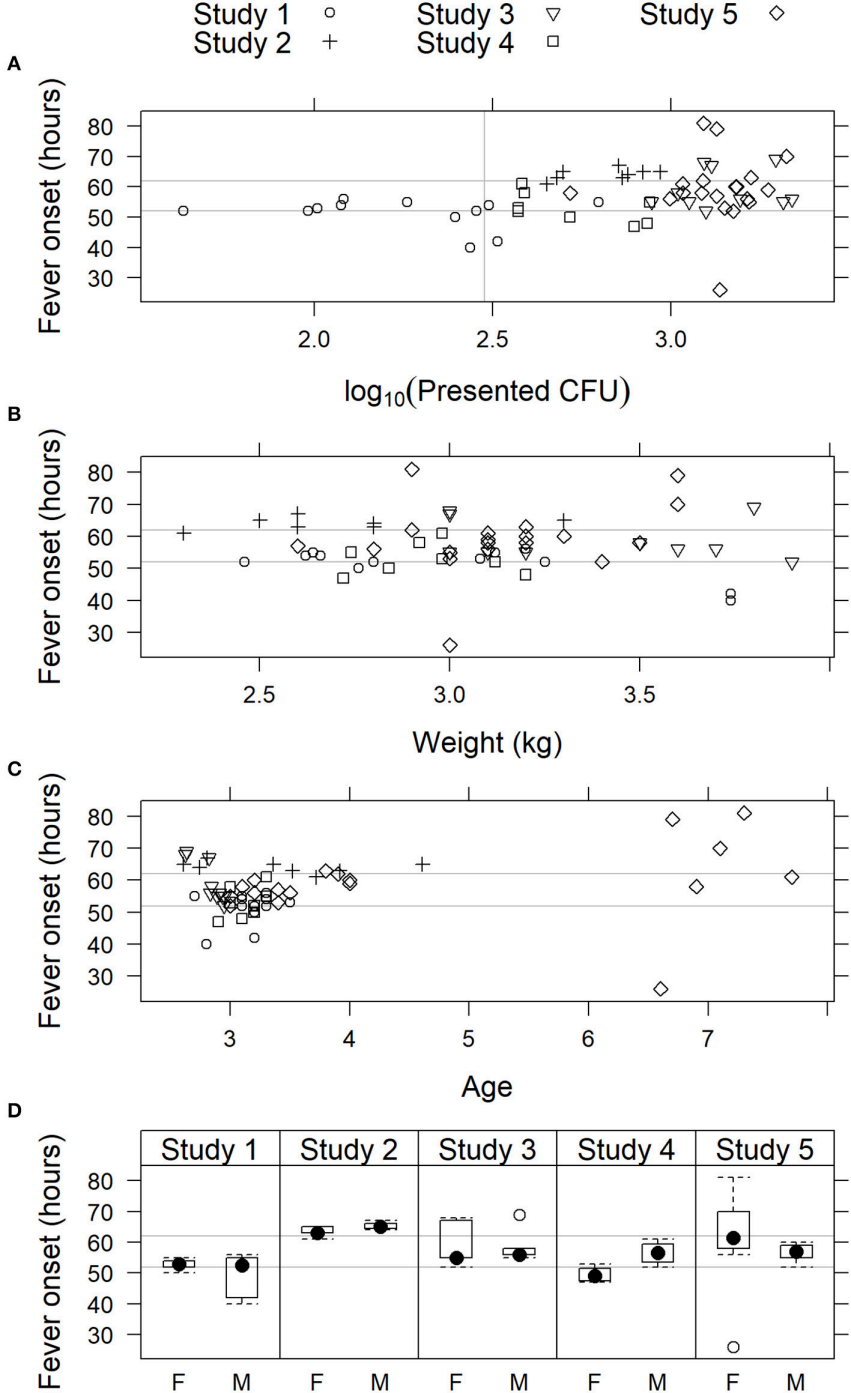
Relationship of fever onset with physiological parameters in cynomolgus macaques exposed to 43 to 2,182 cfu *F. tularensis* Schu S4. In studies where fever onset was modeled, time of onset is plotted vs. **(A)** presented dose of *F. tularensis*; **(B)** body weight; **(C)** age; and **(D)** sex: female (F) or male (M). In (A–C), individual animals are represented by symbols, while sex data is presented as box and whiskers plots. Light gray horizontal lines represent the 95% confidence limits (52 and 62 h) of fever onset across all five studies.

Models that included effect of inhaled *F. tularensis* dose, weight, age, or sex on fever onset were not supported by the data (Figure 4). For the complete data set, AIC was lowest for the model that included age; the simple random effects model was nearly as well supported with ΔAIC = 0.60. However, the effect of age was not strong (coefficient 1.47 h later for each additional year of age, 95% confidence interval −0.33, 3.26). Furthermore, noting the advanced age of seven subjects in Study 5 (aged 6 or 7 years; all 50 other subjects, including the 12 other subjects in that study, were aged 2–4 years), the influence of those older subjects was investigated by fitting the models to the data excluding subjects more than 4 years old. For that truncated data set, AIC is lower for the simple random effects model (model including age ΔAIC = 1.56), meaning the truncated data do not strongly support including an age effect. While the coefficient for age remained positive, its 95% confidence interval broadly overlapped zero (1.26 h later for each additional year of age, 95% CI −2.52, 5.07). The age effect appeared spurious. Also for the complete data set, AIC for the model that included a fixed effect of inhaled dose was only 1.11 AIC units higher than the simple random effects model; however, the coefficient of the former model was positive (1.84 implying longer time to fever for higher inhaled doses) and its confidence interval included zero (−2.27, 4.67). These results showed that there was no strong effect of inhaled *F. tularensis* dose (43–2,182 cfu range), animal weight, age, or sex on fever onset in the studies presented herein.

### Onset of hypothermia and morbidity

**Figure 5:**
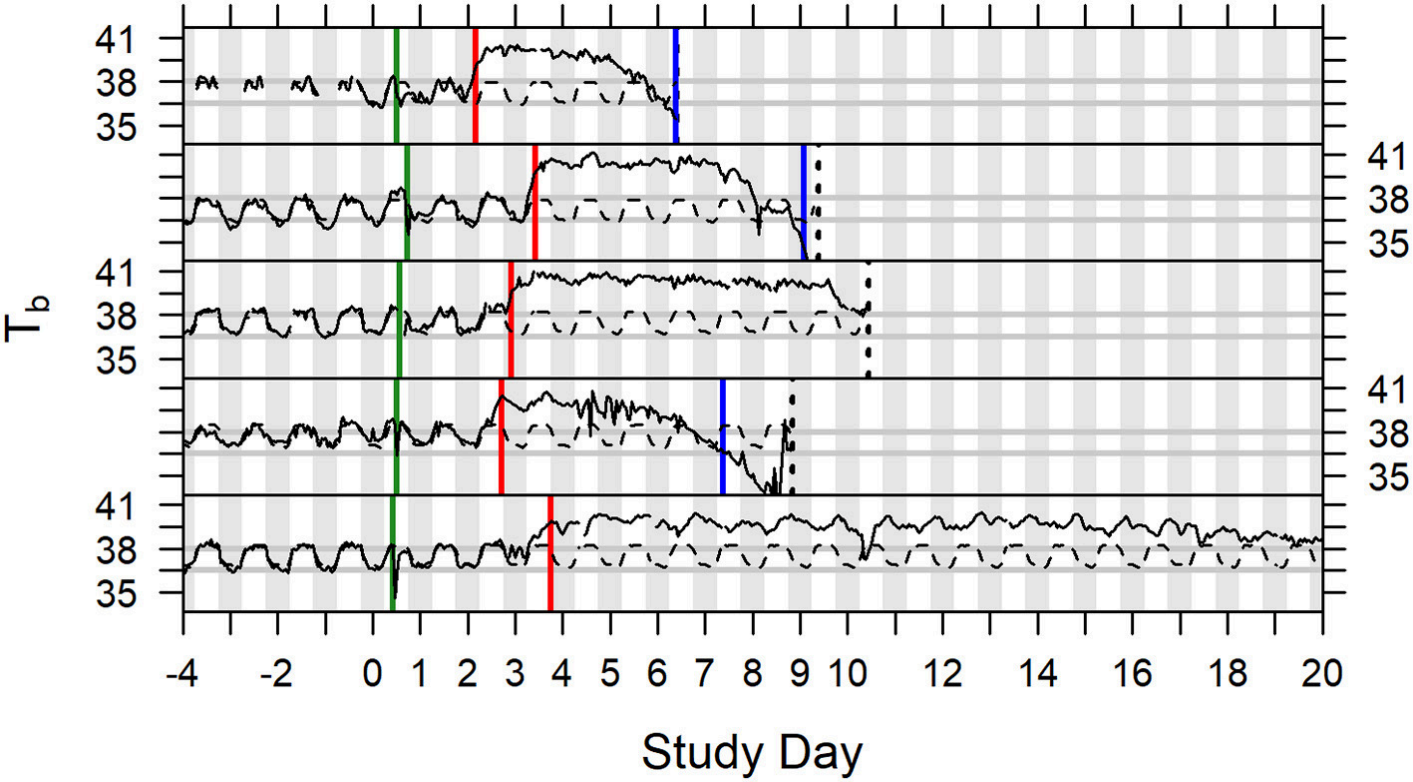
Time series plots of body temperature for five animals depicted with subject specific model predictions of circadian rhythm, Study Days −4 through 19. Green vertical lines indicate exposure to aerosolized *F. tularensis* on Study Day 0. Red vertical lines indicate onset of fever. Blue vertical lines indicate onset of hypothermia. Dashed black vertical lines represent the time of death (the fifth subject illustrated survived to the end of the study).

In studies described herein, animals were euthanized when they exhibited at least one protocol-defined euthanasia criterion, or at the scheduled termination of the study. Hypothermia was not included in protocol-defined euthanasia criteria in any of the six studies described. Onset of hypothermia was defined retrospectively after all studies were completed. Body temperature recorded by telemetry (Studies 1–5; Study 6 had incomplete data) was modeled to evaluate hypothermia as a tool for determining morbidity. Thirty-three (33) of the 57 subjects (Studies 1 through 5) were determined to have gone into hypothermia (Table S1), which was the first instance when temperature depression was ≤-1.5°C over two or more consecutive hourly averages. For five (5) subjects, time of hypothermia coincided with time of death (hypothermia determination at or 1 h before last telemetry recording). Twenty-six (26) other subjects survived for 3–57 h after hypothermia onset with steadily declining or irregular low body temperature (generally staying at least 1°C below subject-specific baseline) without ever recovering warmth. That left two subjects with indication of recovering warmth following hypothermia determination. In one case, body temperature rose above the subject's baseline again that evening through the next 4 days, even with periods of fever the following night, but it did not warm up on the third day when it was euthanized; that subject had unusually low baseline and was retrospectively flagged for large variance of residuals in the pre-exposure period. A second exception subject, which had not sustained fever more than about 24 h, regained warmth the morning after the hypothermia determination but then had low body temperature the following afternoon and was euthanized the day after that. A subset of animals (10 animals of 57; 17.5%) in all the analyzed studies (Studies 1 through 5) exhibited euthanasia criteria that indicated the onset of morbidity and mortality without meeting the criteria for onset of hypothermia. Examples of core body temperature variation and onset of fever and hypothermia in individual animals are presented in Figure 5. Based on this analysis, we propose using hypothermia as a tool for determination of morbidity and inclusion of hypothermia in euthanasia criteria.

### Pathology

#### Macroscopic findings

The macroscopic findings observed during necropsy examination were similar regardless of the dose received, and/or the time-to-death following *F. tularensis* exposure. However, those animals receiving the lowest doses generally had less severe lesions affecting fewer organs. The most common lesions were observed in the lung, lymphoid tissues (spleen and lymph nodes), and liver.

Pulmonary findings typically affected all lung lobes, however, the caudal (inferior) lung lobe often appeared to have more severe changes. Findings in the lung included (but were not limited to) enlargement (failure to collapse), edema, mottled dark reddish-purple to black discoloration, fibrin on pleura, and randomly distributed, well-circumscribed to coalescing, tannish-white, firm to fluctuant foci. Additional thoracic findings include: blood-tinged, straw-colored pericardial fluid, and mediastinal edema.

Macroscopic findings in lymphoid tissues included enlargement and discoloration of lymph nodes and spleen and white/tan foci in the spleen. The most commonly affected lymph nodes were the mediastinal and tracheobronchial lymph nodes however similar lesions were observed in the mandibular, axillary, inguinal, and mesenteric lymph nodes. Splenic changes were also common but varied in severity between animals. The most noteworthy macroscopic lesion in the spleen consisted of tannish-white, flattened to slightly raised foci randomly dispersed throughout the parenchyma and capsular surface.

Hepatic changes included mild enlargement, dark red/brown color often with an accentuated lobular pattern. In some animals, randomly distributed, tannish-white foci were seen on the hepatic surface.

#### Histopathology

It is important to note that the tissue list for microscopic evaluation was different for each study. The only tissues evaluated microscopically in all animals from all studies were brain, liver, lung, tracheobronchial/bronchial lymph node, and spleen. In addition, the number and/or location of routine sections of lung processed to slide for microscopic evaluation were different for studies from each laboratory. Each laboratory handled the processing of pulmonary gross lesions in different ways.

In general, animals that received the lowest challenge doses had less severe lesions affecting fewer organs and tissues. The most common lesions were observed in the thoracic cavity, in the lung and mediastinal and tracheobronchial lymph nodes. Lesions were similar but varied in severity consisting of bronchiolar and alveolar inflammatory infiltrates of neutrophils and macrophages along with exudation of fibrin and protein-rich fluid, necrosis of infiltrating cells and pulmonary parenchyma, hypertrophy/hyperplasia of alveolar macrophages, and thrombi. Lesions were often associated with larger conducting airways and arterioles as well as randomly distributed, discrete inflammatory nodules which effaced alveolar and bronchiolar architecture. The latter was more common especially as the disease progresses. Pleural infiltrates with neutrophils and macrophages were also observed along with exudation of fibrin and protein-rich fluid and necrosis of infiltrating cells and pleural parenchyma. Lymphatic, perivascular and mural vascular infiltrates of neutrophils, macrophages, and exudation of fibrin were frequently observed along with necrosis of infiltrating cells. When present, these changes were also associated with perivascular edema and hemorrhage.

Hematopoietic and lymphoid tissues available for microscopic evaluation had inflammatory infiltrates, necrosis, hemorrhage, and/or edema similar to that observed in the lung. Affected lymph nodes had multifocal to coalescing areas of inflammatory infiltrates (neutrophil and macrophage), as well as edema and lymphoid follicle depletion. Infiltrating cells and lymphoid tissues were often in various stages of degeneration and necrosis. Lesions in the spleen included micronodular inflammatory infiltrates (neutrophil and macrophage), necrosis of red pulp and infiltrating cells, red pulp/lymph follicle depletion, and/or variable inflammation. As the disease progressed, splenic inflammation was accompanied by microthrombi in the splenic sinuses and trabecular vessels. In the most severely affected spleens, there was widespread necrosis of the red pulp and apoptosis and depletion of lymphocytes in the white pulp. In the bone marrow, micronodular inflammatory infiltrates (neutrophil and macrophage) were observed typically with necrosis of the infiltrating cells. In some animals, these infiltrates were accompanied by hemorrhage and myeloid hyperplasia.

The presence of micronodular inflammatory infiltrates was the primary change observed in affected livers from challenged animals. Inflammatory infiltrates (neutrophil and macrophage) were randomly distributed throughout the hepatic parenchyma with necrosis of infiltrating cells and in some cases adjacent hepatocytes. Inflammatory infiltrates were often accompanied by the presence of apoptotic cells in the hepatic sinusoids (likely hepatocytes, Kupffer cells) and/or intrasinusoidal fibrin microthrombi. Occasionally necrosis of small groups of hepatocytes and Kupffer cells were observed near sinusoids but not adjacent to areas of inflammatory infiltrate accumulations.

Few changes were observed in the kidney of challenged animals. These included hemorrhage, renal tubular degeneration, and foci of inflammatory infiltrates (neutrophils and macrophages) of minimal severity and low incidence.

Congestion and/or hemorrhage in the adrenal gland, pancreas, the urinary bladder mucosa, and throughout the gastrointestinal tract, when they occurred and were examined microscopically, were likely due to systemic *F. tularensis* infection. The most noteworthy gastrointestinal tract lesions were observed on the mucosal and, to a lesser extent, serosal surfaces of the pyloric stomach, ileocecal colic junction, and distal colon, suggestive of gastrointestinal-associated lymphoid tissue (GALT) hyperplasia, necrosis/inflammation, and/or hemorrhage.

**Figure 6:**
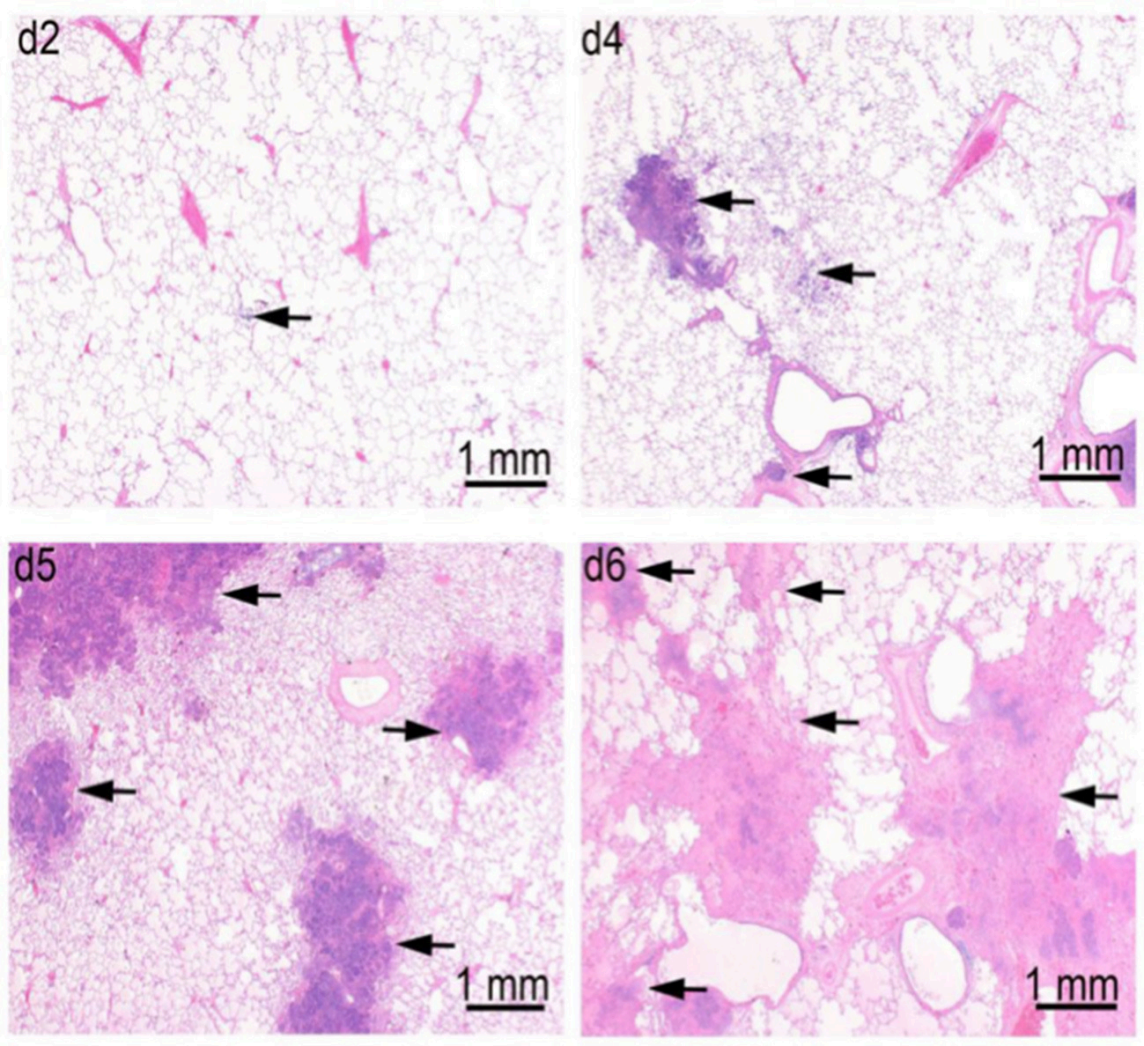
Progression of lung lesions in cynomolgus macaques on Days 2 (d2), Day 4 (d4), Day 5 (d5), and Day 6 (d6) after challenge with aerosolized *F. tularensis* Schu S4. Data is shown for animals in the pathogenesis progression arm of Study 1 (Table 1). Arrows point to foci of inflammation and necrosis.

In a pathogenesis progression arm of Study 1 (Table 1), the progression in severity of lesions in the lung, lymph nodes, spleen, and liver was clearly demonstrated. Pulmonary lesions progressed in severity from Day 2 through Day 6 post-challenge (Figure 6). Pulmonary lesions identified on Day 2 consisted of minimal to mild alveolar inflammatory infiltrates (neutrophil and macrophage), with fibrin exudation into the bronchioles and peribronchiolar alveoli. By Day 4, lesions had increased in severity, and were accompanied by inflammatory infiltrates of vessels (perivascular and mural), bronchioles, lymphatics, and pleura. By Days 5 and 6, all lesions increased in severity, and there was evidence of necrosis of infiltrating inflammatory cells and pulmonary parenchyma, with increased fibrin exudation and edema into alveoli. Inflammatory infiltration of the pleura become extensive, and no longer only associated with the parenchymal foci.

Lymph nodes were affected in a similar manner with tracheobronchial and retropharyngeal lymph nodes affected first and most severely, followed by the mandibular lymph nodes. Initial lesions include micronodular inflammatory infiltrates of neutrophils and macrophages with fibrin exudates. By Days 5 and 6, necrosis of infiltrating cells and parenchyma had become so severe that some lymph nodes were left with only stromal framework. Comparable lesions were detected in the axillary lymph nodes by Days 5 and 6 in two animals, but no lesions were detected in inguinal and mesenteric lymph nodes. Inflammatory infiltrates in the red pulp of the spleen were observed on Day 2 and progressed to micronodules of neutrophils and histiocytes with necrosis by Day 4. By Days 5 and 6, these nodules expanded with increasing necrosis by Day 5 and 6. In some animals, foci of microthrombi formation in the splenic sinuses were observed at the latter time points. Liver lesions were not evident in any animals until Day 4, when randomly distributed foci of inflammatory infiltrates were observed. These micronodular lesions were more abundant by Day 5 and two animals developed widespread foci of sinusoidal microthrombi in the livers on Days 5 and 6.

A summary of tularemia-associated microscopic findings in the lungs of cynomolgus macaques challenged with aerosolized *F. tularensis* Schu 4 in natural history studies is shown in Table 3. In summary, of the tissues available for microscopic evaluation, changes were noted in the lung (inflammatory infiltrates, fibrin, hemorrhage, edema, and/or necrosis), spleen (inflammatory infiltrates, fibrin, hemorrhage, and/or necrosis), lymph nodes (inflammatory infiltrates, necrosis, fibrin, edema, and/or hemorrhage), kidney (inflammatory infiltrates), sternal bone marrow (myeloid hyperplasia, inflammatory infiltrates and/or necrosis), and liver (inflammatory infiltrates and/or necrosis). Microscopic evaluation of tissues from animals in a pathogenesis progression study arm provided support for the progression of lesions in severity and incidence over time. Pulmonary inflammatory infiltrates comprised of neutrophils and macrophages, hemorrhage, fibrin exudation, necrosis (infiltrating cells, pleura, alveoli, bronchioles, vessels), and/or edema were prominent and present in the majority of animals from all studies. In affected animals, inflammatory infiltrates, hemorrhage, fibrin exudation, and/or necrosis were often observed in the thoracic lymph nodes (tracheobronchial and/or mediastinal) consistent with spread of bacteria from lymphatic drainage of infected pulmonary tissue. These lesions occurred in other lymph nodes (mandibular, axillary, mesenteric, and inguinal lymph nodes) but less frequently.

**Table 3:** Summary of tularemia-associated microscopic findings in the lungs of cynomolgus macaques challenged with aerosolized *F. tularensis* Schu 4 in five natural history studies.

**Number examined**	Study 1^*^	**Study 2**	**Study 3**	Study 4^*^	**Study 6**	**Total**
	**12^a^**	**9^b^**	**10**	**8**	**32**	**71**
Congestion	0	0	0	2	0	2
Edema, interstitium, intra-alveolar, and/or perivascular	1	9	10	8	31	59
Fibrin, intra-alveolar	0	8	10	5	30	53
Hemorrhage	12	2	0	2	20	36
Hyperplasia, bronchiolar	0	0	0	0	0	0
Inflammation, necrotizing, alveoli/bronchiole	0	2	0	0	0	2
Inflammation, necrotizing, vessels/perivascular	0	2	0	0	0	2
Inflammatory infiltrates, alveoli/bronchiole/bronchi, neutrophil, and macrophage^c^	12	7	10	8	30	67
Inflammatory infiltrate, intra-alveolar, macrophage	12	8	10	7	30	67
Inflammatory infiltrate, intra-alveolar, neutrophil	12	8	10	7	30	67
Inflammatory infiltrates, vessels/perivascular/mural, neutrophil, and macrophage	12	7	10	8	30	67
Necrosis, alveoli/bronchiole/bronchi	0	0	0	1	0	1
Pleura, inflammatory infiltrate, neutrophil, and/or macrophage with/without fibrin	11	9	10	9	25	64
Within normal limits	0	0	0	0	0	0

* *Includes data from the pathogenesis progression arms of Studies 1 and 4 (see Table 1)*.

a *Includes one animal that survived to study termination (Day 22)*.

b *One animal was euthanized 2 days post-aerosol challenge due to complications from the telemetry unit surgery not due to illness from the challenge material. Therefore, the microscopic findings from this animal are not included in this summary table*.

c *In many sections presented for microscopic evaluation, necrosis of infiltrating cells and pulmonary parenchyma are prominent centrally with surrounding macrophages and exudated fibrin*.

Microscopic evidence of septicemic spread of *F. tularensis* included nodular inflammatory infiltrates in the spleen (often accompanied by hemorrhage, fibrin exudation, and/or necrosis), liver (inflammatory infiltrates, necrosis and/or neutrophilic infiltrate), and sternal bone marrow (myeloid hyperplasia, micronodular inflammatory infiltrates, and/or necrosis).

### Comparison of pneumonic tularemia in cynomolgus macaques and humans

Clinical signs of pneumonic tularemia in cynomolgus macaques exposed to a target dose of 1,000 cfu (range 300–3,000 cfu) aerosolized *F. tularensis* Schu S4 and human pneumonic tularemia cases from published literature are highly similar (Table 4). In naturally occurring human cases, the infection route, time and dose are typically unknown and can only be estimated. For this reason, time to disease onset in human cases and animal studies is calculated from different starting points. The most frequently documented and typical clinical signs in both humans and the cynomolgus macaques were fever, detection of *F. tularensis* in blood or various organs (including lung, hilar lymph nodes, spleen, or liver), and change in heart and respiratory rates. Bacteremia was typically detected later than clinical signs and fever onset in both species (Table 4 and Table S1). It is difficult to compare bacteremia onset and bacterial burden after exposure to *F. tularensis* in human cases vs. animal models. In reported human cases, *F. tularensis* exposure doses, aerosolization parameters and exact time of exposure prior to onset of symptoms were unknown. In the studies reported herein, cynomolgus macaques were exposed to high doses of *F. tularensis* delivered in small particle aerosol, which resulted in severe disease and bacterial dissemination. Bacteremia in cynomolgus macaques exposed to 300–3,000 cfu aerosolized Schu S4 was observed starting on Day 4 after exposure (see Table S1), mean day of death at 8.6 days post-exposure, whereas onset of fever, the first consistent sign of disease, occurred by Day 2–3 post-exposure. Bacteremia is likely a later disease stage event in both cynomolgus macaque model and human pneumonic tularemia disease cases. Bacteremia would not be detectable in patients soon after the antibiotic treatment is initiated, which may have also contributed to the low detection rates in human cases.

**Table 4:** Natural course of pneumonic tularemia in human and cynomolgus macaque.

**Disease symptoms and signs Human^a^**		**Cynomolgus macaque^b^**
Time course of disease	Approximately 2 weeks from onset of symptoms to death, with a range of 10–25 days	Typically 2–7 days from onset of fever to death
Body temperature	Fever can develop after a few days of illness (i.e., after 2–3 days after inhalation of a high dose of *F. tularensis* Schu S4)	Fever in 100% of cases (typically starting on Day 2–3 post-exposure)
*F. tularensis* detected	Positive in blood but not in all cases, positive in pharyngeal washings, sputum specimens, and gastric aspirates	Positive in blood but not in all cases, positive in lung, liver, spleen, and lymph nodes
Heart rate	Usually elevated but it can be slower than would be expected in the presence of high fever (pulse – temperature deficit)	Elevated (typically starting on Day 2 or 3 post-exposure)
Respiration rate	No change initially. Fulminant disease can rapidly progress to pneumonia and respiratory failure	Elevated (typically starting on Day 2 or 3 post-exposure)
Lung pathology	Pleural exudates, adhesions, and focal modular lesions can be found. Lobular pneumonia often involving all lobes is observed with areas of coagulation and caseous necrosis and sometimes cavitation. Microscopically, the exudate is composed of mononuclear cells with few lymphocytes, erythrocytes, epithelial cells, and plasma cells. The alveolar spaces are filled with exudate and sometimes fibrin. The alveolar septa are congested and may be necrotic. Blood vessels may show mononuclear infiltration, necrosis, and thrombosis. The perivascular lymphatics may be distended with a cellular or caseous exudate	Adhesions and discoloration of the lungs; fluid in the thoracic and abdominal cavities; necrotizing inflammation with variable amounts of hemorrhage and edema. The lesions were most consistent with a subacute necrotizing and suppurative bronchopneumonia with the most extensive lesions seen associated with larger airways and pulmonary arterioles and arteries. Abundant macrophages were present within neutrophilic or necrotic foci in the alveoli of lungs or surrounding liquefied necrotic centers forming caseating granulomas
Other findings	No specific clinical laboratory findings stand out. White blood count may reveal leukocytosis but not as elevated as would be expected for invasive bacterial disease. Increased CRP levels	Moderate leukocytosis on Day 2–3 followed by a drop after 48 h. Increase in CRP levels starting on Day 3

a *Data from publications describing pneumonic tularemia in humans (Permar and Maclachlan, 1931; Blackford and Casey, 1941; Stuart and Pullen, 1945; McCrumb, 1961; Overholt et al., 1961; Saslaw et al., 1961; Hornick and Eigelsbach, 1966; Sawyer et al., 1966; Beisel, 1967; Beisel et al., 1967; Provenza et al., 1986; Syrjala, 1986; Penn and Kinasewitz, 1987; Tarnvik et al., 1989; Hoel et al., 1991; Scofield et al., 1992; Sjostedt et al., 1997; Dennis et al., 2001; Feldman et al., 2001; Haristoy et al., 2003; Lamps et al., 2004; Tarnvik and Chu, 2007; Penn, 2009; Fritzsch and Splettstoesser, 2010; Thomas and Schaffner, 2010; Egan et al., 2011; Weber et al., 2012; Johansson et al., 2014)*.

b *Reference is made to the disease signs in animals exposed to 300–3,000 cfu Schu S4 in natural history studies described in this manuscript*.

## Discussion

Based on the review and analysis of results of the studies described herein, we propose a set of critical parameters for the cynomolgus macaque (*M. fascicularis*) model of pneumonic tularemia to be used in the therapeutic efficacy studies as described in Table 5 and below. These criteria are currently under FDA review and may be revised upon final model qualification. The final qualification statement will be published on the FDA web site.

**Table 5:** Proposed critical parameters for the cynomolgus macaque (*Macaca fascicularis*) model of pneumonic tularemia.

Animal characteristics	Study inclusion criteria	Equal number of experimentally naïve males and female of Indochinese origin
		Aged between 3 and 7 years, weighing ≥2.5 kg
		Healthy based on a clinical veterinary evaluation and history that reveals the absence of any clinically relevant abnormality^a^
	Study exclusion criteria	Any clinically significant (as deemed by the Clinical Veterinarian and Study Director) history of acute illness within 4 weeks of screening^b^
		Having evidence of previous exposure to *F. tularensis* or *Trypanosoma cruzi*
		Use of any antibiotic, antifungal, or antiparasitic within 14 days of challenge with *F. tularensis*
		Positive for a panel of viruses, bacteria, and parasites^c^
		Animals with increased white blood cell (WBC) counts or increased C-reactive protein (CRP) defined as a statistically significant increase over the corresponding reference range^d^
Challenge material	*F. tularensis* Schu S4 cell bank	Documented *F. tularensis* Schu S4 isolate history, provenance, and genomic DNA sequence corresponding to sequence of Schu S4 isolate described herein (BEI Cat. No. NR-10492, NCBI Accession #PRJNA217349)
		Documented cell bank identity, purity, viability, growth curve, and virulence in a small animal model (e.g., mouse, rat, or rabbit)
	Challenge material preparation	Growth in enriched Mueller-Hinton Broth (MHB) or Chamberlain's liquid medium that support *F. tularensis* Schu S4 growth
		Aerosol generator suspension is prepared using bacterial cultures in logarithmic phase of growth that were propagated from colonies of expected morphology
	Challenge material delivery	Delivery of 300–3,000^e^ cfu of *F. tularensis* Schu S4 in 1–5 μm diameter aerosol particles using head-only exposure chamber
		Air volume inhaled by each animal is measured using methods such as plethysmography
		Concentration of viable aerosolized bacteria is measured during the exposure by enumerating the bacteria in an all-glass impinge
		Purity and colony morphology of challenge material delivered to each animal is confirmed
		Environmental conditions including air humidity and temperature during animal exposure are monitored and documented (>60% humidity in the exposure chamber is recommended)

*Table contains critical parameters for the cynomolgus macaque model to be used in therapeutic efficacy studies as proposed by the Tularemia AMQ Working Group. Final model parameters may be modified upon FDA review*.

a *Includes a physical examination, medical history, vital signs, ophthalmologic exam, the results of clinical chemistry, and hematology tests, and a urinalysis carried out within 30 days of challenge*.

b *Including asthma, or presence of cardiovascular, pulmonary, hepatic, renal, hematologic, gastrointestinal, endocrine, metabolic, immunologic, dermatologic, neurologic, or psychological disease; for example as assessed during the physical examination required for quarantine release. Current significant diarrhea, gastric stasis, or constipation*.

c *Positive for Simian T Lymphotropic Virus (STLV-1), Simian Immunodeficiency Virus (SIV), Simian Retrovirus (SRV) Types 1 and 2, Macacine herpesvirus 1 (Herpes B virus), confirmed by currently accepted testing within 90 days of challenge. Positive Salmonella, Shigella, Plasmodium, and intestinal parasites, confirmed by currently accepted testing within 30 days of challenge. A positive TB test within 30 days of challenge*.

d *Upon first recognition of either or both of these laboratory abnormalities, additional veterinary clinical evaluation of the animals including but not limited to additional physical examinations, additional blood draws for CRP- and WBC-value trends over time, WBC differentials and morphology, evaluation of other laboratory parameters (e.g., clinical chemistry) and the magnitude of the increases of CRP and WBC values should be conducted*.

e *Challenge material dose range of 300–3,000 cfu F. tularensis Schu S4 is recommended based on similarities in disease progression and survival rates of cynomolgus macaques that were exposed to this dose range across all studies and performance sites, and the feasibility and the precision of delivery and quantification of 1,000 cfu Schu S4 challenge dose across all studies and performance sites*.

### Animal model inclusion and exclusion criteria

Study animals need to fulfill defined inclusion criteria prior to challenge to be eligible for participation in the study. The following criteria are recommended for use in therapeutic efficacy studies: equal number of experimentally naïve males and females of Indochinese origin, aged between 3 and 7 years, weighing ≥2.5 kg. Animals of ~2 to ~7 years of age have been included in natural history studies described in this manuscript. There was no strong effect of animal sex, age, or weight on fever onset (see Figure 4), and no differences in disease progression or outcomes were observed in animals challenged with 309–2,182 cfu *F. tularensis* Schu S4 in natural history studies. However, we recommend including animals of 3–7 years of age and weighing ≥2.5 kg in therapeutics efficacy studies in which animals are subjected to additional pre-challenge and post-challenge procedures. Two-year old and younger animals are quite small (often <2 kg) which increases both the anesthetic and surgical procedure risks, and decreases the blood volume available for analyses. In addition, smaller animals are at greater risk for dehydration and deteriorating condition because they have limited body reserve capacity.

Animals are to be healthy based on a clinical veterinary evaluation and history that reveals the absence of any clinically relevant abnormality, which includes medical history, absence of viral, bacterial and parasitic infections, a physical examination, vital signs, ophthalmologic exam, the results of clinical chemistry, and hematology tests, and a urinalysis carried out within 30 days of challenge. In addition to clinically significant health issues, animal model study exclusion criteria consider any recent exposure to drugs or previous exposure to infectious agents, including *F. tularensis*, which may affect the course of infection and therapeutic efficacy study outcome. None of the studies included animal prescreening or examination of disease progression by lung radiography. We have aimed to develop and include model characterization methods that may be utilized by multiple research facilities, as some facilities may not have the necessary radiographic equipment in Biosafety level 3 laboratories. However, chest radiography may be considered as additional diagnostic methodology for use in animal prescreening in future studies.

Cynomolgus macaques that were used in the herein reported studies were obtained from Indochina. Recent publications describe the genetic heterogeneity of cynomolgus macaques obtained from Indochina, with some animals showing genetic evidence of interbreeding with rhesus macaques that started during the Pleistocene (Kanthaswamy et al., 2008). The bulk of cynomolgus macaques imported into the United States inhabit this active hybrid zone in Southeast Asia that includes Vietnam, Cambodia, Laos, and Burma. Consequently, Indochinese cynomolgus macaques exhibit much greater genetic diversity than cynomolgus macaques from other geographic locations. Genetic evidence of natural interspecies admixture between cynomolgus and rhesus macaques in Indochina, leading to gene flow well beyond the hybrid zone, is well-documented (Kanthaswamy et al., 2013). This genetic diversity may account for differences in individual animal response and variations in disease and treatment outcomes. However, this model behaves consistently even with this genetic heterogeneity, such that drug efficacy can be extrapolated to a heterogeneous population of humans. In addition, age, weight, and sex of animals challenged with 300–3,000 cfu Schu S4 did not impact fever onset in studies described herein (see Figure 4).

### Challenge material source, preparation, and delivery

To ensure the quality and reproducibility of exposures across the study, *F. tularensis* used for animal challenge is to be prepared from the cell banks generated from an isolate of a known history and provenance. Cell bank manufacture and storage, viability, morphology, and virulence in acceptable animal models of infection should be documented. The genomic DNA sequence of the *F. tularensis* Schu S4 cell bank used in studies described in this manuscript (BEI Cat. No. NR-10492, NCBI Accession #PRJNA217349) is identical, less a SNP, with previously published *F. tularensis* Schu S4 genomic DNA sequence (GenBank number AJ749949.2, Larsson et al., 2005). A recent study comparing virulence of various *F. tularensis* strains in mice suggested that *F. tularensis* Schu S4 is not as highly virulent as *F. tularensis* clinical isolates, including an isolate (MA00-2987, Feldman et al., 2001; Matyas et al., 2007) from the blood of a fatal human case with pulmonary infection on Martha's Vineyard in year 2000 (Molins et al., 2014). We have performed full genome sequencing and compared DNA sequences of NIAID cell bank NR-10492 and the Schu S4 isolate used in the above referenced study (Molins et al., 2014). We also compared virulence of these two *F. tularensis* Schu S4 isolates side by side with the *F. tularensis* MA00-2987 in Fischer 344 rat model of pneumonic tularemia (Hutt et al., 2017). No rats (*n* = 10/group) that were exposed to target dose of 100 cfu aerosolized NIAID cell bank NR-10492 Schu S4 or MA00-2987 survived after 7 days post-exposure, while all rats exposed to aerosolized Schu S4 isolate that was used in Molins et al. (2014) survived through Day 21 post-exposure (manuscript in preparation). In addition, the less virulent Schu S4 isolate (Molins et al., 2014) had nine regions of difference in the genomic DNA sequence when compared to NIAID cell bank NR-10492, which may explain its reduced virulence in the rat model of pneumonic tularemia (manuscript in preparation). These results show that rigorous characterization of cell banks that are the source of infectious material is critical for successful animal model development.

A series of aerosol delivery qualification studies with sham aerosols (all steps taken except the animal exposure) were conducted at three performance sites (BBRC, LRRI, and USAMRIID). These studies showed that delivery of aerosolized *F. tularensis* with acceptable cell viability and spray factor occurred when bacteria were grown to logarithmic phase prior to dilution into an aerosol generator mixture. There was no difference in disease progression and animal survival whether *F. tularensis* Schu S4 was grown in enriched MHB or Chamberlain's medium prior to exposure. *F. tularensis* was delivered in 1–5 μm diameter aerosol particles in the total volume of air that was estimated to contain the target dose of *F. tularensis* based on the minute inspiration volume recorded for each individual animal immediately prior to exposure using plethysmography. Viability and purity of aerosol generator mixture and delivered bacterial dose (samples collected in all-glass impinger) was analyzed by plating on agar after the exposure.

### Animal model endpoints

The Tularemia AMQ WG intent is to qualify the natural history model of pneumonic tularemia in the cynomolgus macaque that will provide the basis for use of the model in evaluating the efficacy of antimicrobial therapeutics for the treatment and/or post-exposure prophylaxis of pneumonic tularemia under the Animal Rule. This model may also be used in the future for the development of vaccines for use in prevention of pneumonic tularemia. The Animal Rule states that the primary animal study endpoint has to be clearly related to the desired benefit in humans, such as the enhancement of survival or prevention of major morbidity (FDA, 2009, 2015). The Tularemia AMQ WG proposes that animal survival at a prospectively defined time point should be the primary endpoint for therapeutic efficacy studies using the cynomolgus macaque model of pneumonic tularemia. This endpoint may differ in further refinement of this model for use in evaluation of vaccines efficacy.

Model secondary endpoints can consist of other critical model parameters/biomarkers that correlate with the disease progression and indicate that the model is functioning as expected across studies. We propose time to fever onset and bacteremia to be used as model secondary endpoints. Fever onset preceded clinical signs in animals across all studies reported in this manuscript. All 57 animals challenged with 300–3,000 cfu aerosolized *F. tularensis* Schu S4 in the natural history or therapeutic efficacy studies described herein exhibited onset of fever within 81 h, therefore, we propose that fever onset within this time frame in studies performed within the above described model parameters would serve as one of the indicators showing that the model is functioning as expected. In addition, we propose that fever onset be used as the trigger for initiation of treatment. Delay of treatment studies in which drug is administered at various time points after the onset of disease (in this case defined as the fever onset) may need to be performed to demonstrate drug therapeutic efficacy in advanced disease models. Hypothermia, defined as body temperature drop below the baseline (see Figure 5 and accompanying text), may be indicative of the onset of morbidity in animal models of tularemia, as it was shown in a study that correlated hypothermia and survival of mice infected by *F. tularensis* (Molins et al., 2014). We propose to use hypothermia as one of the euthanasia criteria in future studies using this model.

Detection of bacteremia that correlates with challenge agent material is a common biomarker that is correlated with bacterial dissemination and disease progression. Unlike in infections with some other bacterial Tier 1 agents, bacteremia has not been consistently detected on specific days after challenge in the cynomolgus macaque model of pulmonary tularemia. This has also been observed in human cases of pulmonary tularemia, likely due to the relatively low levels of *F. tularensis* in blood during acute pneumonic tularemia (Saslaw et al., 1961).

Other potential model secondary endpoints examined by the Tularemia AMQ WG were hematology parameters and CRP levels. Changes in hematological parameters and CRP are non-specific indicators of disease. Results of the natural history studies described herein do not identify any hematological biomarkers of pneumonic tularemia. Although changes in hematology parameters are sometimes seen, none are identified as pathognomonic nor does there appear to be a characteristic pattern of change with pneumonic tularemia. Therefore, the WG believes the utility of these parameters is more applicable to inclusion or exclusion of animals onto therapeutic efficacy studies.

The results and analyses of the above described studies support the conclusion that signs of pneumonic tularemia in cynomolgus macaques exposed to 300–3,000 cfu of aerosolized *F. tularensis* Schu S4, under the conditions described herein, and human pneumonic tularemia cases are highly similar. The proposed *F. tularensis* Schu S4 challenge dose range resulted in disease progression that would allow sufficient time for the initiation of therapeutic treatment after the onset of fever. Therefore, we propose that this model may be used for testing and development of tularemia therapeutics under Animal Rule. This model may also be further developed for use in efficacy testing of tularemia vaccines.

## Author contributions

Conceived and designed the studies: TG, LL, MW, CH, PS, and JH. Analyzed the data: TG, LL, KO, MW, SH, CH, and JH. Wrote the paper: TG, LL, and KO.

### Conflict of interest statement

KO is employed by Mergus Analytics, LLC, a subcontractor to TRI. The other authors declare that the research was conducted in the absence of any commercial or financial relationships that could be construed as a potential conflict of interest.

## Supplementary material

Figure S1 Kinetics of changes in hematology and CRP-values in cynomolgus macaques after exposure to *F. tularensis*. The kinetics of changes in WBC counts **(A)**, neutrophil counts **(B)**, lymphocyte counts **(C)**, and CRP plasma levels **(D)** in Studies 1, 2, 3, and 4 **(Table 1)** were plotted by study day. The data are plotted using a box and whiskers plot (Tukey method), where the dotted red vertical line represents the study day in which most animals developed fever (mean time to fever onset was 57 ± 8 h) and the shaded area for the hematology parameters represent the normal ranges in cynomolgus macaques.

Table S1 Onset of fever, hypothermia and bacteremia. Onset of fever and hypothermia are shown for animals in four natural history studies (Study 1 through 4) and antibiotic efficacy study (Study 5). Bacteremia is shown for Studies 1 through 4.
