# Supplementary figures and images for: The Cynomolgus Macaque Natural History Model of Pneumonic Tularemia for Predicting Clinical Efficacy Under the Animal Rule
Source: Front Cell Infect Microbiol. 2018 Apr 4;8:99. doi: 10.3389/fcimb.2018.00099 (PMC5893833; doi:10.3389/fcimb.2018.00099)

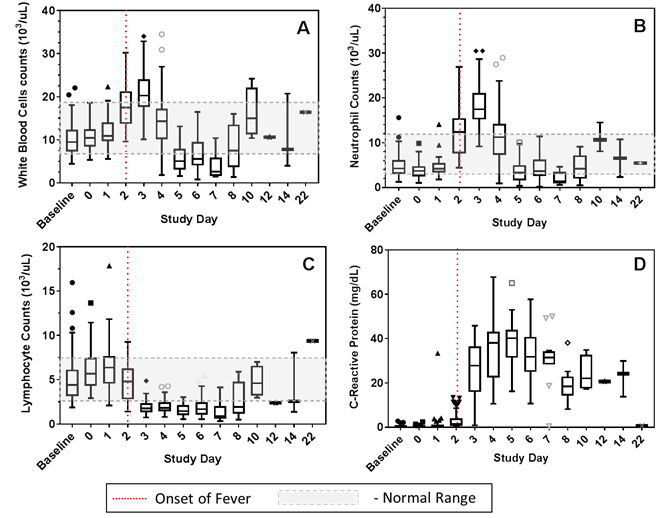

Supplement: Figure S1 — Kinetics of changes in hematology and CRP-values in cynomolgus macaques after exposure to F. tularensis. The kinetics of changes in WBC counts (A), neutrophil counts (B), lymphocyte counts (C), and CRP plasma levels (D) in Studies 1, 2, 3, and 4 (Table 1) were plotted by study day. The data are plotted using a box and whiskers plot (Tukey method), where the dotted red vertical line represents the study day in which most animals developed fever (mean time to fever onset was 57 ± 8 h) and the shaded area for the hematology parameters represent the normal ranges in cynomolgus macaques. [file Image1.TIF]
