# Supplementary material for: The Cynomolgus Macaque Natural History Model of Pneumonic Tularemia for Predicting Clinical Efficacy Under the Animal Rule
Source: Front Cell Infect Microbiol. 2018 Apr 4;8:99. doi: 10.3389/fcimb.2018.00099 (PMC5893833; doi:10.3389/fcimb.2018.00099)
Supplement: Table S1 — Onset of fever, hypothermia and bacteremia. Onset of fever and hypothermia are shown for animals in four natural history studies (Study 1 through 4) and antibiotic efficacy study (Study 5). Bacteremia is shown for Studies 1 through 4. [file Table1.DOCX]

**Table S1. Onset of fever, hypothermia and bacteremia.**

Onset of fever and hypothermia are shown for animals in four natural history studies (Study 1 through 4) and antibiotic efficacy study (Study 5). Bacteremia is shown for Studies 1 through 4.

| **Study Number** | **Animal Number** | **Animal Sex** | **Presented Dose (cfu)** | **Fever Onset (hrs)** | **Hypothermia Onset (hrs)** | **Time to Death (hrs)** | **Bacteremia on agar (Study Day)** | **Bacteremia qPCR (Study Day)** |
| --- | --- | --- | --- | --- | --- | --- | --- | --- |
| 1 | 1 | F | 285 | 52 | 166 | 168 | 4 | NA |
| 1 | 2 | F | 309 | 54 | na | 170 | 2 | NA |
| 1 | 3 | F | 119 | 54 | na | 528 | 3 | NA |
| 1 | 4 | F | 43 | 52 | 148 | 156 | 6 | NA |
| 1 | 5 | F | 182 | 55 | 148 | 152 | 4 | NA |
| 1 | 6 | F | 248 | 50 | na | 152 | 4 | NA |
| 1 | 7 | M | 96 | 52 | 171 | 168 | 4 | NA |
| 1 | 8 | M | 102 | 53 | 173 | 182 | 4 | NA |
| 1 | 9 | M | 121 | 56 | na | 188 | 8 | NA |
| 1 | 10 | M | 627 | 55 | 147 | 148 | 5 | NA |
| 1 | 11 | M | 327 | 42 | na | 136 | 5 | NA |
| 1 | 12 | M | 273 | 40 | 141 | 142 | 4 | NA |
| 2 | 13 | F | 835 | 65 | 159 | 198 | 5 | NA |
| 2 | 14 | F | 448 | 61 | 162 | 210 | 5 | NA |
| 2 | 15 | F | 932 | 65 | 156 | 193 | 5 | NA |
| 2 | 16 | F | 731 | 63 | 152 | 192 | 6 | NA |
| 2 | 17 | F | 479 | 63 | 158 | 171 | 4 | NA |
| 2 | 18 | M | 714 | 67 | na | S | ND | NA |
| 2 | 19 | M | 758 | 64 | 201 | 208 | 6 | NA |
| 2 | 20 | M | 498 | 65 | 116 | 175 | 5 | NA |
| 3 | 21 | F | 2063 | 55 | na | 331 | 5 | 4 |
| 3 | 22 | F | 1124 | 55 | na | 334 | ND | 5 |
| 3 | 23 | F | 1251 | 52 | na | 335 | ND | 3 |
| 3 | 24 | F | 1301 | 67 | 305 | 331 | 4 | 4 |
| 3 | 25 | F | 1237 | 68 | na | 333 | 6 | 4 |
| 3 | 26 | M | 1044 | 58 | 284 | 305 | 7 | 3 |
| 3 | 27 | M | 1970 | 69 | na | 332 | 5 | 4 |
| 3 | 28 | M | 1566 | 56 | 190 | 210 | 6 | 4 |
| 3 | 29 | M | 2182 | 56 | na | 237 | ND | 4 |
| 3 | 30 | M | 884 | 55 | 265 | 266 | 5 | 3 |
| 4 | 31 | M | 373 | 52 | na | 153 | 5 | NA |
| 4 | 32 | F | 521 | 50 | 158 | 175 | 4 | NA |
| 4 | 33 | M | 388 | 58 | 264 | 310 | 5 | NA |
| 4 | 34 | M | 382 | 61 | na | S | ND | NA |
| 4 | 35 | M | 872 | 55 | 146 | 166 | 4 | NA |
| 4 | 36 | F | 857 | 48 | 98 | 141 | 5 | NA |
| 4 | 37 | F | 373 | 53 | 165 | 200 | 4 | NA |
| 4 | 38 | F | 789 | 47 | 147 | 188 | 4 | NA |
| 5 | 39 | F | 1079 | 61 | 189 | 202 |  |  |
| 5 | 40 | F | 1234 | 81 | 357 | 368 |  |  |
| 5 | 41 | F | 2109 | 70 | 382 | 403 |  |  |
| 5 | 42 | F | 1081 | 58 | na | 838 |  |  |
| 5 | 43 | F | 1219 | 58 | na | 838 |  |  |
| 5 | 44 | F | 1370 | 26 | 75 | 184 |  |  |
| 5 | 45 | F | 1344 | 79 | na | 839 |  |  |
| 5 | 46 | M | 1518 | 60 | 230 | 262 |  |  |
| 5 | 47 | M | 1872 | 59 | na | 838 |  |  |
| 5 | 48 | F | 1230 | 62 | na | 838 |  |  |
| 5 | 49 | F | 1675 | 63 | na | 837 |  |  |
| 5 | 50 | M | 1415 | 53 | na | 838 |  |  |
| 5 | 51 | F | 1637 | 56 | na | 838 |  |  |
| 5 | 52 | M | 1344 | 57 | na | 838 |  |  |
| 5 | 53 | M | 521 | 58 | 170 | 179 |  |  |
| 5 | 54 | M | 993 | 56 | na | 168 |  |  |
| 5 | 55 | M | 1528 | 60 | 145 | 164 |  |  |
| 5 | 56 | M | 1657 | 55 | 187 | 203 |  |  |
| 5 | 57 | M | 1495 | 52 | na | 839 |  |  |

Cfu, colony forming units; hrs, hours; F, female; M, male; na, not applicable, animals succumbed before hypothermia onset; S, animal survived challenge; ND, not detected; NA, not analyzed. Data for Study 5 (shaded cells) will be presented elsewhere.
